# The congruency of neuropsychological and F18-FDG brain PET/CT diagnostics of Alzheimer’s Disease (AD) in routine clinical practice: insights from a mixed neurological patient cohort

**DOI:** 10.1186/s12883-022-02614-4

**Published:** 2022-03-09

**Authors:** Sascha Hansen, Jana Keune, Kim Küfner, Regina Meister, Juliane Habich, Julia Koska, Stefan Förster, Patrick Oschmann, Philipp M. Keune

**Affiliations:** 1 Department of Neurology, Klinikum Bayreuth GmbH, Hohe Warte 8, 95445 Bayreuth, Germany; 2 Institute of Physiological Psychology, University of Bamberg, Bamberg, Germany; 3 Department of Nuclear Medicine, Klinikum Bayreuth GmbH, Bayreuth, Germany

**Keywords:** Neuropsychological diagnostics, Cognition, Alzheimer ‘s disease, FDG-PET/CT

## Abstract

**Background:**

Diagnostics of Alzheimer’s Disease (AD) require a multimodal approach. Neuropsychologists examine the degree and etiology of dementia syndromes and results are combined with those of cerebrospinal fluid markers and imaging data. In the diagnostic process, neuropsychologists often rely on anamnestic and clinical information, as well as cognitive tests, prior to the availability of exhaustive etiological information. The congruency of this phenomenological approach with results from FDG-PET/CT examinations remains to be explored. The latter yield highly accurate diagnostic information.

**Method:**

A mixed sample of *N* = 127 hospitalized neurological patients suspected of displaying a dementia syndrome underwent extensive neuropsychological and FDG-PET/CT examinations. Neuropsychological examinations included an anamnestic and clinical interview, and the CERAD cognitive test battery. Two decisional approaches were considered: First, routine diagnostic results were obtained, i.e. the final clinical decision of the examining neuropsychologist (AD_Clinical_ vs. non-AD_Clinical_). Secondly, a logistic regression model was implemented, relying on CERAD profiles alone. CERAD subscales that best predicted AD based on FDG-PET/CT were identified and a nominal categorization obtained (AD_Test_ vs. non-AD_Test_). Congruency of results from both approaches with those of the FDG-PET/CT (AD_PET_ vs. non-AD_PET_) were estimated with Cohen’s Kappa (κ) and Yule’s Y coefficient of colligation. Descriptive estimates of accuracy, sensitivity and specificity of CERAD relative to FDG-PET/CT diagnostics were derived.

**Results:**

AD_PET_ patients constituted *N* = 33/127 (26%) of the sample. The clinical decision approach (AD_Clinical_ vs. non-AD_Clinical_) showed substantial agreement with the FDG-PET/CT classification (κ = .69, Y = .72) involving good accuracy (84.2%), moderate sensitivity (75.8%) and excellent specificity (92.6%). In contrast, the decisional approach that relied on CERAD data alone (AD_Test_ vs. non-AD_Test_) involved only moderate agreement with the FDG-PET/CT (κ = .54, Y = .62) with lower accuracy (74.8%), attributable to decreased sensitivity (56.3%) and comparable specificity (93.3%).

**Conclusions:**

It is feasible to identify AD through a comprehensive neuropsychological examination in a mixed sample of neurological patients. However, within the boundaries of methods applied here, decisions based on cognitive test results alone appear limited. One may conclude that the clinical impression based on anamnestic and clinical information obtained by the neuropsychological examiner plays a crucial role in the identification of AD patients in routine clinical practice.

**Supplementary Information:**

The online version contains supplementary material available at 10.1186/s12883-022-02614-4.

## Background

Alzheimer’s disease (AD) is considered to be the most common type of dementia, with prevalence rates drastically increasing from below 1% for patients aged 65 to 74 to above 22% for patients aged 85 and older [1]. Cognitive decline, in particular episodic memory impairment, is the leading and most debilitating symptom of AD with an onset early in the disease [2]. Valid tools to assess cognitive deficits are neuropsychological test batteries such as the Consortium to Establish a Registry for Alzheimer’s Disease Neuropsychological Assessment Battery (CERAD-NAB) [3]. However, since cognitive deficits constitute a common symptom in many neurological diseases, the specificity of generalized cognitive decline when differentiating between different forms of dementia is inevitably negligible. In order to successfully differentiate between different forms of dementia, clinical symptoms have to be assessed in closer detail. This is feasible via neuropsychological test batteries such as the CERAD-NAB, that assess cognitive deficits in several domains such as attention, memory, executive function, language, motor praxis and visuo-spatial perception. The validity of this approach has been evaluated positively in numerous studies [4–6], although some limitations have also been reported [7]. Neuropsychologists can usually rely on additional clinical information regarding anamnesis, behavioral monitoring during the administration of the neuropsychological tests and information from family members or next-of-kin. These may also serve as an important source of information when determining the most likely diagnosis, albeit they may not always be systematically obtained or reported during routine clinical practice [8, 9].

Neuroimaging techniques, on the other hand, have become a complementary approach to differential diagnostics with increasing accuracy. Concerning the etiological differentiation between AD and non-demented patients, FDG-PET/CT may play an important role in establishing a diagnosis, as its’ predictive value has been determined as excellent in this regard [10]. Moreover, FDG-PET/CT also has the ability to differentiate between different types of dementia with a high accuracy [11, 12]. FDG-PET/CT represents a minimally invasive imaging procedure that measures the regional distribution of cerebral metabolic glucose. Specific patterns of regional glucose reduction allow inferences about the etiology of the underlying disease [13].

In routine clinical practice, it remains to be determined in detail, which aspects of the neuropsychological assessment contribute to the accuracy of the differential diagnostic decision. In recent studies, sensitivity and specificity of neuropsychological diagnostics reached reasonably high values [5, 6], but these results were based on information from both, neuropsychological assessments as well as additional information gained during the clinical process. On the other hand, research suggests that specific deficit profiles exist that may allow a differentiation based on test results alone [4, 14]. For instance, in AD, episodic memory decline is reported to be the leading cognitive symptom at least in the early stages of the disease [2].

Based on these considerations, the purpose of the current study was to examine the congruency of neuropsychological diagnostics and FDG-PET/CT diagnostics, in terms of differentiating between AD versus non-AD patients in a mixed neurological sample. With regards to the neuropsychological diagnostics, two decisional approaches were implemented: The first approach was a clinical decision approach (AD_Clinical_ vs. non-AD_Clinical_). This approach considered information from the standardized neuropsychological assessment as well as all additional unstandardized anamnestic and behavioral information gleaned during the neuropsychological examination. In essence, this approach reflects the final decision of the clinical neuropsychologist as it occurred in routine clinical practice. The second approach relied on a logistic regression model, based on the neuropsychological test results alone (AD_Test_ vs. non-AD_Test_). For both decisional approaches, diagnostic reliability and colligation relative to FDG-PET/CT diagnostics were estimated. To provide further descriptive information, results of both decisional approaches were also matched against the FDG-PET/CT diagnostic results in cross-tables to derive estimates of accuracy, sensitivity and specificity. Even though FDG-PET/CT diagnostics do not represent a *gold standard* in differential diagnostics of neurodegenerative diseases, such estimates may provide further valuable descriptive information on the congruency of the diagnostic methods considered in the current work and may hence be of value for the concurrent literature and future studies. It was assumed that both decisional approaches would yield sufficient reliability, colligation and predictive values in terms of the differential diagnosis (AD vs. non-AD), relative to the FDG-PET/CT diagnosis. It was further expected that results of the logistic regression model would be in line with the extant literature on characteristic cognitive deficit profiles in AD. Nevertheless, it was also assumed that results of the regression model would be inferior to the more comprehensive clinical approach in differentiating between AD and non-AD patients. The latter hypothesis implies that the overall clinical impression of the neuropsychologist, that incorporates anamnestic information, test results and behavioral monitoring during test administration, is of high relevance for the differential diagnostic decision.

## Methods

The CERAD-NAB was administered to 127 inpatients at the Department of Neurology, Klinikum Bayreuth GmbH, Germany, during the routine clinical process. Recruitment occurred consecutively during the routine process throughout the project’s funding period between 10/2018–10/2020. Testing was conducted by highly practiced psychologists, specialized in the field of neuropsychology. In sum, five psychologists with an average post-graduate work experience in the field of neurology of M = 5.4 years were engaged in obtaining data for the current project. This occurred under continuous supervision of two clinical neuropsychologists (SH, PMK), licensed in Germany according to the German Society of Neuropsychology (GNP) and authorized for the implementation of clinical training according to GNP regulations (SH).

Neuropsychological assessments included an unstandardized interview to obtain basic anamnestic information. During the interview, the psychological staff also explored subjective cognitive deficits during activities of everyday life, with the intention to differentiate between AD-relevant deficits in episodic memory, versus deficits in attention and executive functioning. Further, psychological strain due to potential cognitive deficits and the relevance of such deficits for functioning in everyday life was explored. This included particularly the issue, whether living independently was still possible. In addition, it was considered whether patients displayed a behavioral tendency to trivialize or minimize observable memory deficits in their subjective reports, suggesting anosognosia or anosodiaphoria, both of which represent relevant clinical features in AD [15]. The characteristics above were not formally assessed by means of a standardized assessment tool, but were explored in context of a brief anamnestic and clinical interview, reflecting the routine clinical process, of approximately 15–30 min duration. The clinical impression formed during this interview provided a basis for the interpretation of results of the subsequently implemented neuropsychological examination by means of the CERAD.

Following the neuropsychological examination, patients underwent an extensive neurological assessment including neuroimaging diagnostics via FDG-PET/CT. Inclusion criteria involved suspected cognitive decline observed upon admission to the hospital that resulted in a referral to the department of neuropsychology for further testing, as it was assumed to be compatible with the presence of a syndrome of dementia according to ICD-10 [16], i.e. impaired memory and other cognitive functions, unclouded consciousness, as well as a deterioration in motivation, social behavior or emotional control. The etiology of cognitive decline had to be unclear at the time of hospitalization. Patients’ age had to fall in the age range covered by CERAD-norms (49–92 years). Patients were not eligible for inclusion if they had severe visual or hearing impairments that interfered with cognitive testing, or if they had less than 8 years of formal education. They were also not eligible for study entry if they lacked the capacity to consent to participate due to cognitive decline. A flow diagram detailing patient recruitment can be reviewed in Additional file 1. All participants provided written informed consent prior to study entry. The study was approved by the ethics committee of the University of Bamberg, Germany (reference number 2019–02/10). Demographic and clinical information of the sample is displayed in Table 1.

**Table 1:** Demographic and clinical information of the study sample

Groups	AD	non-AD			non-AD subgroups			*p*-value
			PD/PDD	LBD	VD	Unconspicuous	Other	AD vs. non-AD
Participants (N)	33	94	27	16	14	15	22	
Sex (N: male/female)	18/15	53/41	16/11	13/3	6/8	8/7	10/12	.856
Age (years: M, SD)	76.70, 8.29	74.33, 8.36	73.81, 7.95	77.94, 4.91	77.93, 7.09	77.40, 6.07	67.95, 9.48	.163
Education (years: M, SD)	11.09, 1.91	12.53, 2.98	11.89, 2.72	14.44, 3.35	11.14, 2.18	12.80, 2.93	12.64, 2.95	.011*
MMSE (score: M, SD)	23.76, 4.01	25.30, 3.66	26.00, 2.71	22.13, 4.51	25.57, 3.69	27.06, 1.03	25.43, 3.93	.040*

*Note*. Classification of AD versus non-AD and non-AD subgroups based on FDG-PET/CT results. *AD* Alzheimer Disease, *LBD* Lewy Body Dementia, *MMSE* Mini Mental Status Examination, *PD* Parkinson Disease, *PDD* Parkinson Disease Dementia, *VD* ascular Dementia, *significant at *p*-value < .05, two-tailed, based on one-way ANOVA model. The AD group included *N* = 5 cases with mixed etiology, where AD coincided with cerebrovascular disease (*N* = 3), hydrocephalus occlusus (*N* = 1), or where it was classified as atypical AD (*N* = 1). In the non-AD subgroups, the subgroup “other” involved reports of functional anomalies suspected to be of cancerous or post-operative etiology (*n* = 7), progressive supranuclear palsy (*n* = 4), normal pressure hydrocephalus (*n* = 3), multiple system atrophy type C (*n* = 3), frontotemporal dementia (*n* = 2) and unspecified etiology (*n* = 3)

### Neuropsychological assessment

The German version of the CERAD-NAB [3] (CERAD-Plus [6]) was implemented for the neuropsychological examination. The CERAD-Plus consists of the nine basic tests included in the CERAD-NAB, as well as three additional tests assessing executive functions. The 12 tests included in the CERAD-Plus assess the following cognitive domains: verbal and nonverbal memory, verbal fluency, language, praxis, orientation, cognitive flexibility, psychomotor speed and visual scanning. For a detailed description of all subtests please see [17].

### Brain PET/CT imaging procedure

All patients underwent 18F-FDG brain PET/CT scans in a resting state. A mean of 181 MBq F18-FDG was administered intravenously under standardized conditions in a quiet, dimly lit room with patients’ eyes open. The procedure took place in a neuroimaging center experienced with PET (Department for Nuclear Medicine, Klinikum Bayreuth GmbH, Germany). A 10-min 3D-listmode PET emission scan was acquired at mean 40 min post injection in one bed position using a state-of-the-art Siemens Biograph mCT PET/CT scanner with extended field of view (Siemens Healthineers, Erlangen, Germany). During the scanning procedure, patients’ heads were immobilized using a head holder. Attenuation correction was performed using low-dose CT (100 mAs, 120 kV, Collimation 40 × 0.6 mm) before the PET emission scan. Following corrections for scatter, dead time, and random coincidences, PET images were reconstructed by using 3-dimensional filtered back projection, Gaussian Filter, FWHM 3.5 mm. Finally, reconstructed PET data was post-processed on a syngo Via workstation (VB10) using the MI Neurology application. Images were clinically analyzed by a reader experienced in neuroimaging. Analyses took place slice-wise, surface projections-wise, including comparisons with an age-matched, scanner-matched healthy control group, as well as PET/MR fusion-image wise (after coregistration of PET and MRI data and fusion of PET and T2w FLAIR brain MR images).

### Statistical analysis

With regards to the FDG-PET/CT diagnostics, the primary diagnosis (AD_PET_ vs. non-AD_PET_), as noted in the clinical report, was obtained for each participant for the subsequent statistical analysis. Cases in which a mixed etiology was identified, were included in the primary statistical analysis as well, based on the assigned AD diagnosis. However, to ensure that results remained unaffected by this procedure, in a secondary analysis, cases with mixed etiology were excluded and the statistical analysis was repeated.

In order to obtain information about the congruency of results of the neuropsychological examination and the FDG-PET/CT diagnostics, two approaches were implemented. The first one considered a decision model, in which the clinical neuropsychological diagnoses were based on the comprehensive final decision of the examining neuropsychologist. This model hence included the clinical decision whether AD was present or not (AD_Clinical_ vs. non-AD_Clinical_), based on anamnestic information obtained during the examination, behavioral observation during testing, as well as the evaluation of the test results themselves. The congruency of the dichotomous results of this decision model relative to the dichotomous FDG-PET/CT diagnostic results (AD_PET_ vs. non-AD_PET_) was estimated by means of Cohen’s Kappa (κ). In essence, this parameter reflects an estimation of the chance-corrected agreement between the two implemented assessment methods, i.e. neuropsychological examination and FDG-PET/CT examination (inter-rater reliability). Based on original suggestions by Landis & Koch [18], the following nomenclature for the interpretation of Cohen’s κ, i.e. strength of agreement, was used: < 0.00 poor, 0.00–0.20 slight, 0.21–0.40 fair, 0.41–0.60 moderate, 0.61–0.80 substantial, 0.81–1.00 almost perfect. A known limitation of Cohen’s κ is that it is reduced if the base rates of categorical decisions differ across raters [19]. It has been suggested to address this potential issue by the inclusion of Yule’s Y, i.e. a coefficient of collation that is relatively unaffected by base rates [20]. Consequently both, Cohen’s κ and Yule’s Y were used as estimates in the current analyses with the same nomenclature as described above. Analyses were repeated and both parameters Cohen’s κ and Yule’s Y were derived again after the exclusion of cases with mixed etiology of dementia syndromes. To generate further descriptive information, cross-tabulations were used to obtain estimates of sensitivity, specificity and accuracy. To account for a potential asymmetric distribution in the classification of cases, a balanced accuracy statistic was computed as the mean of sensitivity and specificity estimates.

In the second approach, neuropsychological diagnoses were based on the test results alone (AD_Test_ vs. non-AD_Test_) irrespective of the comprehensive clinical diagnosis. In this context, a logistic regression model was implemented. This model included all CERAD subscales as independent variables and the AD_PET_ vs. non-AD_PET_ categorization as the dependent variable. All variables were entered in a single step and the respective non-significant predictor with the lowest significance was removed recursively until solely significant predictors were left in the model. Based on this final model, a cross tabulation was generated and the resulting dichotomous classification was used to generate Cohen’s κ and Yule’s Y relative to the FDG-PET/CT results. Also in case of this test-based model, descriptive estimates of sensitivity, specificity and accuracy were derived.

Finally, a factor analysis was implemented to gain an impression of the convergent validity of the cognitive test results with previous work [21]. All calculations were executed with SPSS 20.0.

## Results

### Sample characteristics

Demographic and clinical characteristics are displayed in Table 1. The sample consisted of 127 patients, 33 of which were diagnosed with AD according to the results of the FDG-PET/CT. As displayed, the sample included diverse etiologies associated with cognitive decline, that were pooled into the two groups AD_PET_ vs. non-AD_PET_. One-way ANOVAs showed that these two groups did not differ significantly in age or sex, but that the AD-group had significantly fewer years of education and also a significantly lower mean score on the Mini Mental Status Examination (MMSE). The latter was a part of the CERAD test battery. Among the *N* = 33 AD_PET_ cases, there were *N* = 5 cases with mixed etiology, where AD coincided with cerebrovascular disease (*N* = 3), hydrocephalus occlusus (*N* = 1), or where it was classified as atypical AD (*N* = 1). A descriptive overview of performance on CERAD subtests and comparisons between AD and non-AD groups is presented in Table 2.

**Table 2:** Comparison of performance on CERAD subtests (z-scores) across diagnostic groups

CERAD parameter	AD	non-AD	*p*-value
	(M ± SD)	(M ± SD)	
BNT	−0.77 ± 1.51	−0.21 ± 1.23	.037*
Wordlist Total	− 2.02 ± 1.34	−1.57 ± 1.55	.138
Wordlist Delayed Recall	−2.25 ± 1.38	−1.15 ± 1,20	.000***
Wordlist Savings	−2.20 ± 1.55	−0.69 ± 1,79	.000***
Wordlist Discrimination	− 2.10 ± 1.56	−1.07 ± 1.54	.001***
Figure Drawings	− 0.61 ± 1.61	−0.60 ± 1.56	.982
Figure Recall	−2.03 ± 1.18	−1.39 ± 1.38	.020*
Figure Savings	−1.93 ± 1.05	−1.12 ± 1.24	.001***
Wordlist Trial 1	−1.25 ± 1.13	−1.01 ± 1.14	.294
Wordlist Trial 2	−1.65 ± 1.13	−1.18 ± 1.47	.091
Wordlist Trial 3	−1.97 ± 1.38	−1.50 ± 1.72	.157
Animals	−0.95 ± 0.97	−1,03 ± 1.26	.747
S-Words	−0.01 ± 1.08	−0.60 ± 1.32	.024*
TMT-A	− 1.14 ± 1.45	−1.18 ± 1.64	.889
TMT-B	−1.70 ± 1.00	−1.54 ± 1.48	.564

Note. Z-scores represent raw test scores relative to the normative database of the CERAD; *M* mean, *SD* standard deviation. *p < .05, **p < .01, ***p < .001 (two-tailed). Note that significant differences between groups emerged predominantly for verbal and nonverbal memory performance

### Congruency of neuropsychological and FDG-PET/CT diagnostic results: clinical decision model

Classification agreement between FDG-PET/CT diagnostics and the neuropsychological clinical decision model was substantial, κ (127) = 0.69, SE = 0.07, approximate T = 7.78, *p* < .001. When controlling for different base rates across classification methods, agreement improved slightly and remained substantial, Y (127) = 0.72. When cases with mixed etiology were excluded, results remained virtually unchanged, κ (122) = 0.70, SE = 0.08, approximate T = 7.76, *p* < .001, Y (122) = 0.74. Descriptive estimates of sensitivity, specificity and overall accuracy for the clinical decision model are displayed in Table 3. Based on the comprehensive neuropsychological examination, 25 patients were diagnosed with AD, out of 33 patients who were categorized as such based on the FDG-PET/CT examination (sensitivity: 75.8%). Out of the remaining 94 patients who were categorized as non-AD patients based on the FDG-PET/CT examination, 87 patients were categorized accordingly based on the neuropsychological examination (specificity: 92.6%). Accuracy was estimated at 84.2%.

**Table 3:** Congruency of the comprehensive neuropsychological and FDG-PET/CT diagnostic results

	FDG-PET/CT Diagnosis		
	AD	Non-AD	
Neuro-psychological Diagnosis			
AD	25	7	32
	Sensitivity: 75.8%		
Non-AD	8	87	95
		Specificity: 92.6%	
	33	94	127
			BA: 84.2%

Note. Cross-tabulation with respective number of cases (total *n* = 127). Displayed are results of the clinical decision approach in which the neuropsychological diagnosis incorporated anamnestic information obtained during the examination, behavioral observation during testing, as well as the evaluation of the test results themselves. *AD* Alzheimer’s disease, *BA* balanced accuracy

### Congruency of neuropsychological and FDG-PET/CT diagnostic results: logistic regression model

Classification agreement between FDG-PET/CT diagnostics and the neuropsychological regression model that was based on the neuropsychological test results alone (AD_Test_ vs. non-AD_Test_) was moderate, κ (121) = 0.54, SE = 0.09, approximate T = 6.02, *p* < .001. When controlling for different base rates across classification methods, agreement improved, Y (121) = 0.62. When cases of mixed etiology were removed from the analysis, results remained virtually unchanged, κ (116) = 0.53, SE = 0.10, approximate T = 5.77, *p* < .001, Y (121) = 0.61.

In the regression model, respective recursive elimination of CERAD variables with the least significant contribution yielded four final variables with a significant contribution to the classification of patients in relation to the FDG-PET/CT diagnostics. Details of the recursive analytical steps are provided in Additional file 1. The four remaining variables included the total immediate recall performance on the episodic verbal memory task of the CERAD (wordlist total), as well as the delayed recall on the same task (wordlist delayed recall). Additionally, recall performance on the episodic non-verbal memory task remained as a significant predictor, specifically the difference between immediate and delayed recall performance on this task, i.e. memory trace decay (figure savings). Hence, as would be expected in context of differential diagnostics in AD, the indicated parameters were associated with the domain of episodic memory for new verbal and non-verbal information. The final fourth variable reflected performance on a phonematic fluency task of spontaneous word generation (s-words). The latter reflects executive functioning, specifically divergent verbal problem solving and overall cognitive speed.

The resulting model with these four variables (wordlist total, wordlist recall, figure savings, s-words) explained 39.5% of variance according to Nagelkerke’s *R*^2^, which reflects a moderate explanatory value. As depicted in Table 4, out of 32 patients categorized as AD patients based on the FDG-PET/CT, 18 were correctly identified based on this model. This reflected a low estimate of sensitivity of 56.3%. On the other hand, estimates of specificity based on this model were excellent, as 93.3% of the non-AD patients categorized through FDG-PET/CT were correctly identified based on the regression model. Accuracy reached a value of 74.8%.

**Table 4:** Congruency of the neuropsychological diagnostics solely based on CERAD test results and the FDG-PET/CT diagnostics

	FDG-PET/CT Diagnosis		
	AD	Non-AD	
Neuro-psychological Diagnosis			
AD	18	6	24
	Sensitivity: 56.3%		
Non-AD	14	83	97
		Specificity: 93.3%	
	32	89	121
			BA: 74.8%

Note. Cross-tabulation with respective number of cases (total *n* = 121), displays results of the test-based decision approach in which the neuropsychological diagnosis was based on the logistic regression analysis of performance on the CERAD test battery alone. Note that the total number of included cases is slightly lower than in the clinical decision approach shown in Table 3, as some patients could not sufficiently draw figures due to motor impairment and consequently these cases were not available for the logistic regression analysis. *AD* Alzheimer Disease, *BA* balanced accuracy

In sum, classification agreement based on Cohen’s κ and Yule’s Y, as well as descriptive accuracy, were reduced for the classification based on the regression model, relative to the clinical decision model. While estimates of specificity were comparable, the logistic regression model involved lower estimates of sensitivity (clinical approach: 75.8% versus test-based approach: 56.3%), that yielded lower overall accuracy of the logistic regression model.

### Exploratory factor analysis

In a post hoc analysis, cognitive test results of the CERAD were subjected to a factor analysis. The factor analysis was implemented to gain an impression of the convergent validity of the cognitive test results. Previous work on the German version of the CERAD has shown that the CERAD involves a three-factor structure and that results from verbal versus non-verbal episodic memory scales of the CERAD load on separate factors [21]. In the current work, the analysis of the underlying factor structure was conducted with a principal factor analysis using promax rotation. The structural matrix of the rotated solution is displayed in Table 5. All calculations were performed using z-scores of the respective CERAD scales. Five parameters were excluded from the analysis as their measure of sampling adequacy (MSA) was below .5, indicating that they were unsuitable for inclusion into the factor analysis (Boston Naming Test, Figure copy, Wordlist Trial 1, Trail-Making-Test A, Trail-Making-Test B/A). After exclusion of these five variables, the Bartlett-test was highly significant (*p* < .001) and the Kaiser-Meyer-Olkin-criterion reached a value of .808, which can be considered good. Both results point to the suitability of the data for executing a factor analysis. Three factors with eigenvalues greater than 1 accounted for 71.22% of variance (Factor 1: 46.87%, Factor 2: 12.97%, Factor 3: 11.38%). Correlations of respective variables with factors suggested that the first factor reflected general verbal cognitive abilities, including verbal fluency and verbal short-term memory. The second factor reflected aspects of nonverbal memory and variables loading on the third factor reflected aspects of verbal memory. In sum, a three-factor structure emerged. Congruent with such a factor structure previously reported for the German version of the CERAD [21], verbal and non-verbal memory functions were distinguishable based on this factor solution.

**Table 5:** Structural matrix of the factor analysis

CERAD Variable		Factor	
	1	2	3
Wordlist Total	.931	.430	.561
S-Words	.710	.277	.036
Animals	.723	.439	−.006
Wordlist Trial 2	.786	.234	.449
Wordlist Trial 3	.918	.412	.481
Figure Recall	.384	.955	.337
Figure Savings	.380	.937	.362
Wordlist Delayed Recall	.680	.452	.881
Wordlist Savings	.210	.295	.844
Wordlist Interference	.032	.048	.388
Wordlist Discrimination	.620	.436	.732
MMSE	.689	.746	.323
TMT-B	.667	.640	.257

Note. Displayed are factor loadings of respective CERAD variables on the three factors labeled as general verbal cognitive abilities (factor 1), nonverbal memory (factor 2) and verbal memory (factor 3)

## Discussion/conclusion

The purpose of the current study was to examine the congruency of neuropsychological differential diagnostics in AD on the one hand and FDG-PET/CT differential diagnostics on the other hand. In terms of the neuropsychological procedure, it was assumed that the comprehensive clinical procedure that relied on anamnestic information, behavioral monitoring during testing and the test results themselves, would show a higher congruency with FDG-PET/CT diagnostics, as compared to a data-driven procedure where the diagnostic decision was based solely on the results of the cognitive tests. The latter hypothesis implies that in routine clinical practice, the overall clinical impression of the neuropsychologist is of high relevance for the differential diagnostic decision.

Results of the current study indicate that the clinical decision approach including a complete neuropsychological workup reached substantial agreement with FDG-PET/CT diagnostics (κ = .69, Y = .72). Further descriptive analyses showed that this agreement was characterized by good accuracy (84.2%) and hence implies sufficient congruency with FDG-PET/CT diagnostics. This is in line with the extant literature concerning the use of the CERAD-NAB in context of comprehensive AD diagnostics [6, 21, 22]. In contrast, the test-based decision approach yielded considerably lower estimates of agreement (κ = 54., Y = .62), as well as lower accuracy (74.8%), the latter attributable to a low sensitivity estimate of only 56.3%. This suggests that about one third of patients with an AD diagnosis based on FDG-PET/CT diagnostics may remain undetected, if only neuropsychological test results are considered. Hence, it may be assumed that obtaining anamnestic information and behavioral monitoring during neuropsychological testing is highly relevant for differential diagnostic decisions in AD patients. Examples of behavioral conspicuities that may show a certain specificity for mild AD and ought to be explored in this context include deficits in episodic memory versus relatively intact attention and executive functioning, combined with a behavioral tendency of patients to trivialize or minimize observable memory deficits in their subjective reports, suggesting anosognosia or anosodiaphoria [15, 23]. Based on the current work, future studies may address the issue of specificity of these conspicuities in more detail.

It is notable that the current results emerged in a mixed, unselected sample including various etiologies that may account for cognitive deficits (Table 1). One might argue that such relatively uncontrolled circumstances may limit the validity of the current findings. On the other hand, the fact that such an unselected, mixed sample was included, might also be regarded as a strength of the current study, as it accurately reflects the actual circumstances in routine clinical practice, as recommended and commonly implemented in Germany [24]. Nevertheless, in consideration of this issue, primary analyses focusing on the congruency of the implemented diagnostic methods were repeated when AD cases with mixed etiology were excluded. As the results remained virtually unchanged, it may be assumed that they remained relatively unaffected by whether an ICD-10 AD diagnosis of F00.0 or F00.2 was given. However, as depicted in Additional file 1, where the flow of patient recruitment is reported, a substantial number of patients who were assigned for the neuropsychological examination had to be excluded from the current study, due to the fact that they were not examined via PET-CT subsequently. In case of these patients, further differential diagnostics via PET-CT were not indicated, as they already displayed moderate to severe cognitive decline. This may be regarded as a selection bias yielding the inclusion of predominantly mild AD patients in the current work. The latter is also illustrated by the still relatively high sample mean score on the MMSE in the AD group (M = 23.76, SD = 4.01; Table 1). Yet this selection bias led to particularly those patients being included, for whom a differential diagnostic procedure including an extensive neuropsychological examination and a PET-CT are of highest relevance. With regards to the sample included in the current work, it also appears noteworthy that several demographic and clinical differences emerged between the AD and non-AD groups, as classified by the FDG-PET/CT (see Table 1). In particular, even though the AD group scored relatively high on the MMSE, this performance was still significantly lower than performance of the non-AD groups. Also, the AD-group differed significantly from the non-AD group on several parameters of the CERAD (see Table 2). Further, AD patients were characterized by significantly fewer years of education. In particular the latter has been described as a potential risk factor for AD [25] and the fact that it emerged as a variable that differed across groups based on the FDG-PET/CT classification is in turn compatible with the notion that the applied FDG-PET/CT diagnostic procedure was methodologically sound.

In a post hoc analysis, a factor analysis was implemented to examine the convergent validity of the neuropsychological test results with those from other studies. In previous work, factor analyses of the CERAD commonly revealed a three-factor solution [21, 26, 27] and the same was the case for data of the current work. Albeit factor labeling may not be entirely consistent across studies, in particular the differentiation between verbal and non-verbal memory factors emerged as a common feature [21] and was also observable in the current work. These findings are generally compatible with the notion that the implementation of the neuropsychological procedure was methodologically sound in the current work. Moreover, it should be noted that in the logistic regression model of the test-based decision approach, two scales of episodic verbal memory and one scale of episodic non-verbal memory were among the four CERAD scales that predicted the classification of patients, relative to the FDG-PET/CT diagnostics. Hence, particularly episodic memory parameters contributed to this decision model, which is in line with the notion that dementia and particularly AD is characterized by episodic memory deficits [2, 28].

In sum, these results imply that the methodological implementation of the neuropsychological tests was sound. Yet, test results by themselves yielded a considerably lower congruency with FDG-PET/CT examinations than the clinical decision approach, for which behavioral monitoring and anamnestic information were available to the clinician. In essence, these findings highlight the necessity of specialized and experienced staff involved in neuropsychological differential diagnostics of AD. It is their expertise which allows the identification of behavioral abnormalities that – in conjunction with the neuropsychological test results – point towards a likely diagnosis of AD.

Nevertheless, several limitations of the current study ought to be noted. First, even though FDG-PET/CT may be regarded as an accurate method to classify different forms of dementia, it may not provide an entirely error-free differentiation and does not represent a diagnostic gold-standard by itself. Yet, descriptive results of sensitivity, specificity and accuracy of AD neuropsychological relative to AD PET-CT diagnostics may be informative for future work and comparable data analytic approaches have been used before in other indications (see e.g. [29, 30]. Further, the purpose of the current work was to explore the congruency of different approaches to neuropsychological diagnostics in AD with FDG-PET/CT and the research design of the current study sufficiently addressed this issue. Secondly, it should be noted that even though it appears justified to conclude that a comprehensive neuropsychological approach including anamnestic information and behavioral monitoring during testing is superior to diagnostics based on test-results alone, a systematic exploration of the specificity of behavioral conspicuities for AD, as suggested above, remains to be implemented in future work.

## Supplementary Information

**Additional file 1.**

## Data Availability

The data generated and analyzed during the current study are available from the corresponding author on reasonable request.
