# Supplementary material for: The congruency of neuropsychological and F18-FDG brain PET/CT diagnostics of Alzheimer’s Disease (AD) in routine clinical practice: insights from a mixed neurological patient cohort
Source: BMC Neurol. 2022 Mar 9;22:83. doi: 10.1186/s12883-022-02614-4 (PMC8905792; doi:10.1186/s12883-022-02614-4)
Supplement: Supplementary file 1 — Additional file 1. [file 12883_2022_2614_MOESM1_ESM.docx]

Appendix 1. Flowchart describing patient recruitment

**N = 94**

Patients deemed not eligible for study entry

(N = 71 no PET/CT scheduled)

(N = 18 early discharge from hospital)

(N = 5 could not be tested due to severe motor, cognitive or sensory impairment)

**N = 263**

Consecutive inpatients *suffering from cognitive decline of unclear origin* pre-screened

**N = 29**

Patients declined to partake in study

**N = 169**

Patients approached for written informed consent

**N = 13**

Datasets excluded

(N = 12 due to missing data)

(N = 1 removed study consent)

**N = 140**

Patients provided written informed consent

**N = 127**

Patients included into final analysis

Appendix 2

*Stepwise display of recursive elimination of the least significant contributing CERAD subscale in the logistic regression model (FDG-PET/CT classification as dependent variable)*

|  |  | B | SE | Wald | Sig. |
| --- | --- | --- | --- | --- | --- |
|  |  |  |  |  |  |
|  |  |  |  |  |  |
| Step 1 | Boston Naming Test | -.293 | .244 | 1.448 | .229 |
|  | Wordlist Total | .179 | .175 | 1.043 | .307 |
|  | Wordlist Delayed Recall | -.781 | .546 | 2.045 | .153 |
|  | Wordlist Savings | -.166 | .332 | .250 | .617 |
|  | Wordlist Discrimination | .030 | .252 | .014 | .907 |
|  | Figure Drawings | .050 | .241 | .042 | .837 |
|  | Figure Recall | .994 | .910 | 1.192 | .275 |
|  | Figure Savings | -1.518 | 1.024 | 2.198 | .138 |
|  | MMSE | .033 | .257 | .017 | .897 |
|  | Wordlist Trial 1 | .145 | .365 | .158 | .691 |
|  | Wordlist Trial 2 | -.226 | .273 | .687 | .407 |
|  | Wordlist Trial 3 | .172 | .329 | .274 | .601 |
|  | Animals | -.011 | .327 | .001 | .974 |
|  | S-Words | .673 | .314 | 4.586 | .032 |
|  | Trail Making Test A | -.007 | .263 | .001 | .978 |
|  | Trail Making Test B | -.142 | .306 | .217 | .641 |
|  | Constant | -2.619 | .622 | 17.717 | .000 |
|  |  |  |  |  |  |
| Step 2 | Boston Naming Test | -.294 | .241 | 1.499 | .221 |
|  | Wordlist Total | .178 | .175 | 1.042 | .307 |
|  | Wordlist Delayed Recall | -.781 | .546 | 2.044 | .153 |
|  | Wordlist Savings | -.167 | .332 | .253 | .615 |
|  | Wordlist Discrimination | .030 | .250 | .015 | .904 |
|  | Figure Drawings | .048 | .234 | .042 | .838 |
|  | Figure Recall | .996 | .908 | 1.203 | .273 |
|  | Figure Savings | -1.519 | 1.023 | 2.205 | .138 |
|  | MMSE | .032 | .254 | .016 | .899 |
|  | Wordlist Trial 1 | .145 | .364 | .159 | .690 |
|  | Wordlist Trial 2 | -.226 | .273 | .686 | .407 |
|  | Wordlist Trial 3 | .172 | .329 | .275 | .600 |
|  | Animals | -.011 | .327 | .001 | .974 |
|  | S-Words | .670 | .298 | 5.049 | .025 |
|  | Trail Making Test B | -.146 | .279 | .273 | .602 |
|  | Constant | -2.618 | .621 | 17.776 | .000 |
|  |  |  |  |  |  |
| Step 3 | Boston Naming Test | -.295 | .240 | 1.518 | .218 |
|  | Wordlist Total | .178 | .175 | 1.041 | .307 |
|  | Wordlist Delayed Recall | -.780 | .545 | 2.045 | .153 |
|  | Wordlist Savings | -.166 | .331 | .252 | .616 |
|  | Wordlist Discrimination | .031 | .250 | .015 | .902 |
|  | Figure Drawings | .049 | .232 | .045 | .833 |
|  | Figure Recall | .988 | .878 | 1.267 | .260 |
|  | Figure Savings | -1.511 | .989 | 2.333 | .127 |
|  | MMSE | .029 | .241 | .015 | .903 |
|  | Wordlist Trial 1 | .146 | .364 | .159 | .690 |
|  | Wordlist Trial 2 | .226 | .273 | .689 | .406 |
|  | Wordlist Trial 3 | .170 | .321 | .281 | .596 |
|  | S-Words | .668 | .289 | 5.332 | .021 |
|  | Trail Making Test B | -.146 | .279 | .275 | .600 |
|  | Constant | -2.616 | .619 | 17.878 | .000 |
| Step 4 | Boston Naming Test | .288 | .232 | 1.537 | .215 |
|  | Wordlist Total | .174 | .171 | 1.032 | .310 |
|  | Wordlist Delayed Recall | -.771 | .541 | 2.032 | .154 |
|  | Wordlist Savings | -.167 | .330 | .256 | .613 |
|  | Wordlist Discrimination | .034 | .248 | .019 | .892 |
|  | Figure Drawings | .053 | .229 | .054 | .816 |
|  | Figure Recall | .989 | .876 | 1.274 | .259 |
|  | Figure Savings | -1.500 | .983 | 2.329 | .127 |
|  | Wordlist Trial 1 | .150 | .363 | .171 | .680 |
|  | Wordlist Trial 2 | -.225 | .273 | .680 | .410 |
|  | Wordlist Trial 3 | .174 | .320 | .295 | .587 |
|  | S-Words | .670 | .288 | 5.398 | .020 |
|  | Trail Making Test B | -.139 | .272 | .259 | .611 |
|  | Constant | -2.625 | .614 | 18.252 | .000 |
|  |  |  |  |  |  |
| Step 5 | Boston Naming Test | -.279 | .218 | 1.646 | .200 |
|  | Wordlist Total | .168 | .169 | .987 | .320 |
|  | Wordlist Delayed Recall | -.753 | .534 | 1.984 | .159 |
|  | Wordlist Savings | -.172 | .329 | .274 | .600 |
|  | Figure Drawings | .044 | .223 | .040 | .842 |
|  | Figure Recall | 1.014 | .871 | 1.355 | .244 |
|  | Figure Savings | -1.515 | .984 | 2.370 | .124 |
|  | Wordlist Trial 1 | .157 | .362 | .187 | .665 |
|  | Wordlist Trial 2 | -.219 | .270 | .658 | .417 |
|  | Wordlist Trial 3 | .180 | .314 | .329 | .566 |
|  | S-Words | .667 | .282 | 5.604 | .018 |
|  | Trail Making Test B | -.135 | .272 | .248 | .619 |
|  | Constant | -2.617 | .609 | 18.479 | .000 |
|  |  |  |  |  |  |
| Step 6 | Boston Naming Test | -266 | .207 | 1.648 | .199 |
|  | Wordlist Total | .168 | .170 | .981 | .322 |
|  | Wordlist Delayed Recall | -.749 | .535 | 1.958 | .162 |
|  | Wordlist Savings | -.181 | .327 | .306 | .580 |
|  | Figure Recall | 1.086 | .791 | 1.885 | .170 |
|  | Figure Savings | -1.579 | .928 | 2.895 | .089 |
|  | Wordlist Trial 1 | .159 | .362 | .191 | .662 |
|  | Wordlist Trial 2 | -.227 | .267 | .725 | .394 |
|  | Wordlist Trial 3 | .183 | .314 | .340 | .560 |
|  | S-Words | .655 | .276 | 5.636 | .018 |
|  | Trail Making Test B | -.124 | .267 | .217 | .641 |
|  | Constant | -2.610 | .607 | 18.464 | .000 |
|  |  |  |  |  |  |
| Step 7 | Boston Naming Test | -.268 | .207 | 1.682 | .195 |
|  | Wordlist Total | .182 | .166 | 1.211 | .271 |
|  | Wordlist Delayed Recall | -.789 | .525 | 2.253 | .133 |
|  | Wordlist Savings | -.145 | .315 | .213 | .645 |
|  | Figure Recall | 1.020 | .767 | 1.770 | .183 |
|  | Figure Savings | -1.505 | .896 | 2.822 | .093 |
|  | Wordlist Trial 2 | -.217 | .260 | .698 | .403 |
|  | Wordlist Trial 3 | .239 | .286 | .700 | .403 |
|  | S-Words | .654 | .275 | 5.679 | .017 |
|  | Trail Making Test B | -.110 | .265 | .174 | .677 |
|  | Constant | -2.641 | .604 | 19.132 | .000 |
|  |  |  |  |  |  |
| Step 8 | Boston Naming Test | -.269 | .207 | 1.691 | .194 |
|  | Wordlist Total | .172 | .164 | 1.104 | .293 |
|  | Wordlist Delayed Recall | -.764 | .521 | 2.150 | .143 |
|  | Wordlist Savings | -.157 | .314 | .250 | .617 |
|  | Figure Recall | .983 | .764 | 1.652 | .199 |
|  | Figure Savings | -1.501 | .901 | 2.775 | .096 |
|  | Wordlist Trial 2 | -.204 | .258 | .626 | .429 |
|  | Wordlist Trial 3 | .207 | .274 | .566 | .452 |
|  | S-Words | .620 | .258 | 5.767 | .016 |
|  | Constant | -2.575 | .577 | 19.924 | .000 |
|  |  |  |  |  |  |
| Step 9 | Boston Naming Test | -.259 | .206 | 1.589 | .208 |
|  | Wordlist Total | .239 | .101 | 5.612 | .018 |
|  | Wordlist Delayed Recall | -.984 | .299 | 10.836 | .001 |
|  | Figure Recall | .920 | .750 | 1.505 | .220 |
|  | Figure Savings | -1.452 | .891 | 2.656 | .103 |
|  | Wordlist Trial 2 | -.234 | .256 | .839 | .360 |
|  | Wordlist Trial 3 | .244 | .267 | .841 | .359 |
|  | S-Words | .621 | .258 | 5.775 | .016 |
|  | Constant | -2.586 | .578 | 20.009 | .000 |
|  |  |  |  |  |  |
| Step 10 | Boston Naming Test | -.247 | .205 | 1.455 | .228 |
|  | Wordlist Total | .235 | .101 | 5.356 | .021 |
|  | Wordlist Delayed Recall | -.973 | .296 | 10.783 | .001 |
|  | Figure Recall | .934 | .758 | 1.518 | .218 |
|  | Figure Savings | -1.481 | .904 | 2.684 | .101 |
|  | Wordlist Trial 3 | .103 | .218 | .226 | .635 |
|  | S-Words | .607 | .258 | 5.525 | .019 |
|  | Constant | -2.519 | .570 | 19.550 | .000 |
|  |  |  |  |  |  |
| Step 11 | Boston Naming Test | -.230 | .202 | 1.299 | .254 |
|  | Wordlist Total | .227 | .100 | 5.170 | .023 |
|  | Wordlist Delayed Recall | -.920 | .271 | 11.517 | .001 |
|  | Figure Recall | .871 | .735 | 1.407 | .236 |
|  | Figure Savings | -1.398 | .876 | 2.545 | .111 |
|  | S-Words | .658 | .235 | 7.830 | .005 |
|  | Constant | -2.590 | .551 | 22.092 | .000 |
|  |  |  |  |  |  |
| Step 12 | Wordlist Total | .227 | .097 | 5.443 | .020 |
|  | Wordlist Delayed Recall | -.897 | .267 | 11.329 | .001 |
|  | Figure Recall | 1.015 | .743 | 1.869 | .172 |
|  | Figure Savings | -1.665 | .881 | 3.576 | .059 |
|  | S-Words | .610 | .222 | 7.529 | .006 |
|  | Constant | -2.665 | .558 | 22.775 | .000 |
|  |  |  |  |  |  |
| Step 13 | Wordlist Total | .233 | .098 | 5.662 | .017 |
|  | Wordlist Delayed Recall | .915 | .263 | 12.067 | .001 |
|  | Figure Savings | -.541 | .256 | 4.457 | .035 |
|  | S-Words | .648 | .221 | 8.596 | .003 |
|  | Constant | -2.644 | .551 | 23.041 | .000 |
|  |  |  |  |  |  |

*Note.* MMSE=Mini Mental Status Test. For a detailed description of all subtests please see [17]. Note that when it was tested whether age and education functioned as significant predictors in the final step of the regression model in a secondary analysis, years of education emerged as an additional significant predictor (B=-.241, SE=.120, Wald=4.052, p=.044), while all previous variables remained significant. In contrast, age did not function as a significant predictor (p=.796).
